# Broadband Microwave Absorbing Composites with a Multi-Scale Layered Structure Based on Reduced Graphene Oxide Film as the Frequency Selective Surface

**DOI:** 10.3390/ma11091771

**Published:** 2018-09-19

**Authors:** Fang Ye, Changqing Song, Qian Zhou, Xiaowei Yin, Meikang Han, Xinliang Li, Litong Zhang, Laifei Cheng

**Affiliations:** Science and Technology on Thermostructural Composite Materials Laboratory, School of Materials, Northwestern Polytechnical University, Xi’an 710072, China; songchangqing@gmail.nwpu.edu.cn (C.S.); qianzhou@mail.nwpu.edu.cn (Q.Z.); songqiang511@nwpu.edu.cn (X.Y.); hanmeikang@mail.nwpu.edu.cn (M.H.); 597438335@mail.nwpu.edu.cn (X.L.); zhanglt@nwpu.edu.cn (L.Z.); chenglf@nwpu.edu.cn (L.C.)

**Keywords:** microwave absorption, reduced graphene oxide, frequency selective surface, multilayer structure

## Abstract

A broadband microwave absorbing composite with a multi-scale layered structure is proposed, in which a reduced graphene oxide (RGO) film sandwiched between two layers of epoxy glass fiber laminates serves as the frequency selective surface (FSS). RGO films with the desired electrical properties were synthesized directly by hydrothermal reaction, vacuum filtration, and heat treatment without subsequent processing. With the novel layer-by-layer structure ranging from micro to macro scale, the optimized composite exhibits excellent microwave absorption performance with a total thickness of 3.2 mm. Its reflection coefficient (RC) is less than −10 dB in the entire X and Ku band, reaching a minimum value of −32 dB at 10.2 GHz and an average RC of −22.8 dB from 8 to 18 GHz. Enhanced microwave absorption of the composites is achieved through the optimization of layer thickness in the sandwich structure to promote destructive interference. Improved impedance matching by the introduction of FSS along with the polarization and conduction loss of layered graphene films also contribute to the increased absorption.

## 1. Introduction

Extensive use of portable electronic instruments and wireless communication has resulted in a serious electromagnetic (EM) interference pollution. Hence, the design and exploration of high-absorption, broadband and lightweight microwave materials, are highly desired [1,2,3,4,5]. Microwave absorbers with multilayer structures that can be easily tuned by tailoring the individual property of materials, have been extensively studied [6,7,8,9,10]. However, broadening the absorption bandwidth of reflection coefficient (RC) to under −10 dB always comes with the unavoidable increase in thickness of the composites [11,12]. It is still a challenging issue in the design of broadband absorbers with low thickness.

Recently, frequency selective surface (FSS) has been proposed as broadband and thin absorbers through the equivalent circuit mode [13,14]. Loaded with lumped components like resistors and capacitors, FSS is usually fabricated with highly conductive materials such as metals with periodic patterns and specific sheet resistances. However, these materials are usually thick and heavy [15]. Reduced graphene oxide (RGO) is a new and promising electromagnetic microwave absorption material, owing to its unique two-dimensional (2D) structure and remarkable properties, such as low density, high specific surface area, tunable electrical conductivity and abundant functional groups [16,17,18,19]. The tunability and frequency-independent surface impedance of graphene at microwave band have been theoretically investigated and proven feasible as a FSS [20]. Huang et al. designed and fabricated graphene FSS patterns on flexible substrates with an effective absorption bandwidth spanning from 10.4 GHz to 19.7 GHz, which is suitable for radar absorption applications [21]. However, the fabrication process of graphene FSS based composites is complex, time-consuming and expensive. Additionally, FSS is usually on the surface of the composite and directly exposed to the air, which increases the possibility of oxidation and degradation of the microwave absorption.

In this work, a thin and broadband microwave absorber is designed and fabricated by using RGO films with layered structures as FSS patterns in a sandwich structure. This design can not only protect RGO films, but also enhances the microwave absorption by optimizing the effective dielectric constant and enhancing the microwave loss mechanism. RGO films with tunable properties, as the unit cell of FSS, have been fabricated by a simple and cost-effective process involving hydrothermal reaction, vacuum filtration, and heat treatment. Subsequently, they are composited with epoxy glass fiber dielectric laminates to develop sandwich microwave absorbing structure. Based on the finite integration simulations, the electrical property of RGO film and the thicknesses of upper and lower dielectric laminates have been optimized. As a result, these RGO films serving as FSS not only optimize the effective dielectric constant but also increase the polarization and conduction loss of the electromagnetic wave. The layer-by-layer structure improves impedance matching, increases macro heterogeneous interfaces and leads to increased absorption by facilitating destructive interference. The synergistic effect of these factors results in excellent microwave absorption with a thickness of 3.2 mm over a wide absorption bandwidth ranging from 8–18 GHz. The proposed structure is superior to most of the reported composites in terms of performance.

## 2. Simulation and Experiments

### 2.1. Materials

All chemicals were of analytical grade and used without further purification. Graphene oxide (GO) was procured from (XF Nano Materials Tech Co., Nanjing, China). The epoxy glass fiber laminate (FR4) was procured from (Tongwen Rubber and Plastic Co., Shanghai, China).

### 2.2. Design and Simulation 

**Figure 1:**
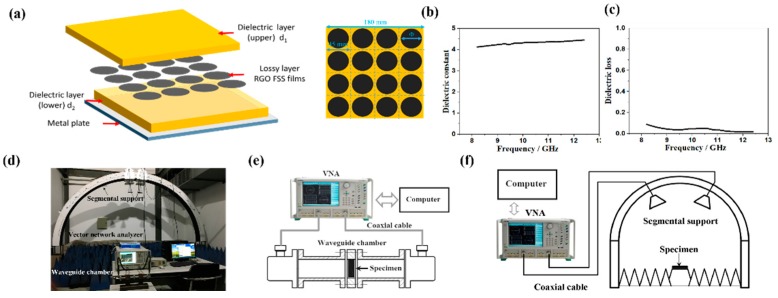
(**a**) Schematic of the fabrication of sandwich-structured microwave absorbing composites; (**b**) the dielectric constant of epoxy glass fiber (FR4) laminate; (**c**) the dielectric loss of FR4 laminate; (**d**) the electromagnetic measurement system used in this study; (**e**) the schematic diagram of the dielectric property test equipment; (**f**) the schematic diagram of the electromagnetic reflection coefficient (RC) test equipment.

The design and optimization of the broadband microwave absorber was performed using the finite integration technique on the CST Microware Studio 2014 package (CST-2015, CST Ltd., Darmstadt, Germany), in which unit cell boundary conditions were applied in the x and y directions. A wave-guide port was used to generate transverse electromagnetic plane waves perpendicularly to the sample plane, which propagates along the −z direction. The absorption could be calculated as A(*ω*) = 1 − T(*ω*) − R(*ω*), where R(*ω*) = |S_11_|^2^ and T(*ω*) = |S_21_|^2^ are the reflectance and transmittance obtained from the frequency dependent complex S-parameters, respectively. Since the backside is grounded by metallic plane, the transmittance T(*ω*) is zero. Thus, the absorption can be reduced to A(*ω*) = 1 − R(*ω*). In the simulation, circular RGO films with a diameter of 39 mm (*Φ*) were selected as the unit absorber cell. They were arranged in a periodic pattern and sandwiched between two FR4 dielectric laminates, as illustrated in Figure 1a. The unit cell was set with periodic boundary conditions in the x–y plane and it was modeled as a resistive sheet with a desired sheet resistance (*Rs*). FR4 laminates were selected as the materials for both the upper and lower substrate, taking impedance match into consideration. The thicknesses of the upper and lower dielectric layers are denoted as *d*_1_ and *d*_2_, respectively. The dielectric constant of FR4 laminate was 4.3 and loss tangent was 0.05 as shown in Figure 1b,c, which can act as a proper electromagnetic impedance matching material.

### 2.3. Preparation of RGO Film (Unit Cell of FSS)

In a typical procedure, 0.2 g of graphene oxide (GO) was well-dispersed in 200 mL of ethylene glycol (EG) (Baishi co. LTD, Tianjing, China) with ultrasonic treatment. The solution was then transferred into a 250 mL Teflon-lined stainless-steel autoclave and heated at 180 °C for 12 h. The RGO colloidal suspension was obtained after the solution was naturally cooled to room temperature. 10 mL RGO suspension was prepared and vacuum filtered with polyvinylidene difluoride (PVDF) membranes (Lesheng filter equipment manufacturing plant, Haining, China) that had a pore size of 0.45 μm and a diameter of 47 mm. Subsequently, the RGO film was fabricated with a diameter of 39 mm and washed with ethanol to remove the remaining solvent. To obtain the highly conductive graphene sheets, RGO films were heat-treated at 600 °C, 800 °C and 1000 °C for 1 h in Ar, marked as RGO-600, RGO-800 and RGO-1000, respectively. RGO films without heat treatment are denoted as RGO-RT.

### 2.4. Preparation of the Composite with Sandwich Structure

**Figure 2:**
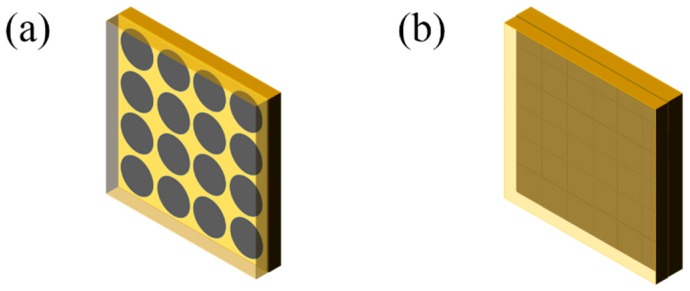
Composites with RGO films in (**a**) periodic array, (**b**) full coverage.

Sixteen of the as-obtained RGO films were arranged periodically and parallel to the surface of the upper and lower FR4 laminates, according to the simulation model in Figure 2a. Four kinds of commercial FR4 laminates with the thicknesses of 1.0 mm, 1.4 mm, 1.8 mm, 2.2 mm and 2.6 mm were used. The dimensions of the composites were 180 mm × 180 mm × *d* (the total thickness of the composite). In case of the RGO composites with full coverage between two dielectric layers, 36 pieces of RGO square films with dimensions of 30 mm × 30 mm formed the composite, as shown in Figure 2b.

### 2.5. Characterization and Measurement 

The morphology of the samples were characterized using field emission scanning electron microscopy (SEM; S-4700, Hitachi, 15 kV, Tokyo, Japan). The crystal phase was identified by X-ray diffraction (XRD, D8 Avance, Bruker, Karlsruhe, Germany) using Cu K*α* (λ = 1.54 Å) radiation (40 kV, 40 mA) in the range of 5–90° with a step scan of 0.01° per step. Raman spectra were acquired using a He-Ne laser (RMS; Renishaw, London, UK) as excitation source (λ = 532 nm). *Rs* of the RGO film was measured using a four-probe equipment (Voganruit technology co. LTD, Company, Beijing, China). The electromagnetic measurement system used in this study was shown in Figure 1d, which was mainly composed of vector network analyzer (VNA, MS4644A, Anritsu, Kanagawa, Japan), waveguide chamber and segmental support. The dielectric property including the real and imaginary part of permittivity of specimens with a plane size of 22.86 mm ×10.16 mm for 8.2–12.4 GHz (X-band) and 15.79 mm × 7.9 mm for 12.4–18 GHz (Ku-band) was measured when VNA was equipped with the waveguide chamber as shown in Figure 1e. The electromagnetic RC of specimens for X and Ku band was measured when VNA was equipped with the segmental support as shown in Figure 1f. Reflection from a pure metal plate with the same size as that of the fabricated composite was used for normalization.

## 3. Results and Discussion

### 3.1. Structural Characterization of RGO Films

**Figure 3:**
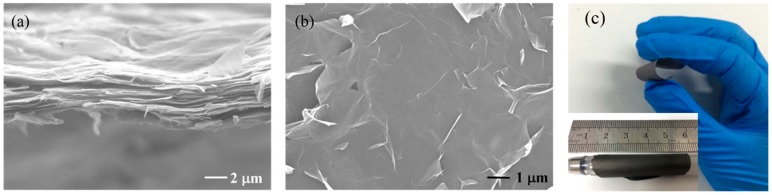
(**a**) Cross-sectional SEM image; (**b**) in-plane SEM image and (**c**) flexibility of the RGO film.

A stacked layer-by-layer structure of the RGO film with thickness under 10 μm is evident from the cross-sectional and in-plane SEM images shown in Figure 3a,b, respectively. The thickness of the RGO film can be tuned by changing the volume of RGO suspension during filtration. This lamellar structure contributes to the absorption of electromagnetic waves that are emit in the vertical direction [22,23,24]. The ultrathin films are flexible as evident from Figure 3c, where they are shown to be bent and rolled around a pen.

**Figure 4:**
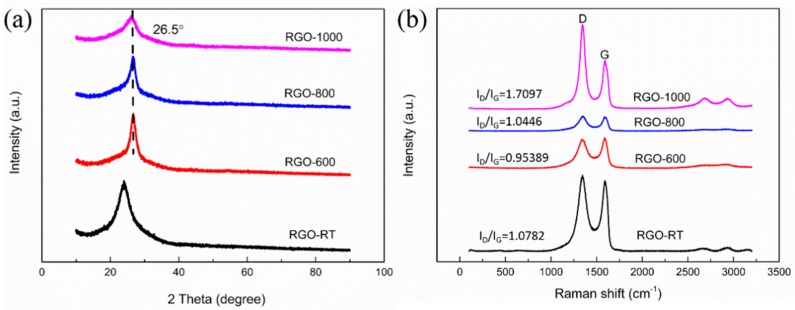
(**a**) XRD and (**b**) Raman spectra of RGO films after different temperature treatments.

Figure 4a reveals the XRD spectra of RGO-600, RGO-800, RGO-1000 and RGO-RT samples. In the case of RGO-600, RGO-800 and RGO-1000, only the graphite crystalline peak is observed in the XRD spectra. The peak at 2*θ* = 26.5° corresponds to the (002) plane of graphite layers with an inter-layer spacing of 0.334 nm [25,26]. For the as-filtrated RGO-RT, a peak around 23.1° indicates a layer-to-layer distance (d-spacing) of about 0.385 nm, which is due to the existence of several oxygen functional groups [27]. Structural characterizations of RGO films are further explored using Raman spectroscopy where two peaks at 1355 cm^−1^ and 1587 cm^−1^ are obtained (Figure 4b), corresponding to the D and G bands, respectively. The peak at 1587 cm^−1^ shows the Raman-active E_2g_ mode or the G band, characterizing the sp^2^ hybridized C–C bonds in RGO. The D band at 1355 cm^−1^ originates from the in-plane vibration of sp^2^ carbon atoms, which reflects the disorder degree of the crystal structure [28,29]. Increased annealing temperature can result in the recovery of the sp^2^ configuration. According to three-stage model from amorphous carbon state to nanocrystalline graphite state, the increase of *I_D_*/*I_G_* from 0.95389 to 1.7097 indicates the increased sp^2^ content [30,31].

For RGO films treated at different temperatures, *Rs* was measured by a four-probe method and the results are listen in Table 1. It is evident that *Rs* decreases dramatically with the increase of annealing temperature. During the heating process from room temperature to 600 °C, the deoxygenation reduction reaction is accompanied by the removal of most of the oxygen functional groups such as –OH, –COOH, from the surface of graphene. Further increasing the temperature from 600 °C to 1000 °C results in a slight decrease in *Rs* due to the slow release of C–H groups and oxygen-containing groups between layers [32].

Based on the simulation results of CST, the composite is optimized to have as wide absorption band when *Rs* of RGO FSS films is 40 Ω/sq. In other words, when RGO-800 films are applied as the interlayer and arranged in a periodic pattern, the composites should exhibit excellent broadband microwave absorption. In this case, RGO-800 films are chosen as the FSS sandwiched by FR4 layers.

### 3.2. Absorption Properties of the Composite with Sandwich Structure

The RC of the composites can be obtained according to transmission line theory:

(1) $${\mathrm{RC}=20\;log}_{10}|\frac{Z_{in}{-Z}_{0}}{Z_{in}{+Z}_{0}}|$$

(2) $$Z_{in}{=Z}_{0}\sqrt{\frac{\mu_{r}}{\varepsilon_{r}}}\tan h[j\frac{2\pi }{c}\sqrt{\mu_{r}\varepsilon_{r}}fd]$$

where, *Z*_0_ = (*μ*_0_/*ε*_0_)^1/2^ is the characteristic impedance of free space, *Z_in_* is the normalized input impedance of absorbing material, *ε_r_* and *μ_r_* are the relative complex permittivity and permeability, respectively. *f* is the frequency, *d* is the thickness of absorbing material, and *c* represents the speed of light in free space. For non-magnetic dielectric substrates, the relative permeability *μ_r_* = 1. When the RC is below −10 dB, more than 90% electromagnetic energy is absorbed and the corresponding frequency range is defined as the effective absorption bandwidth (EAB).

**Figure 5:**
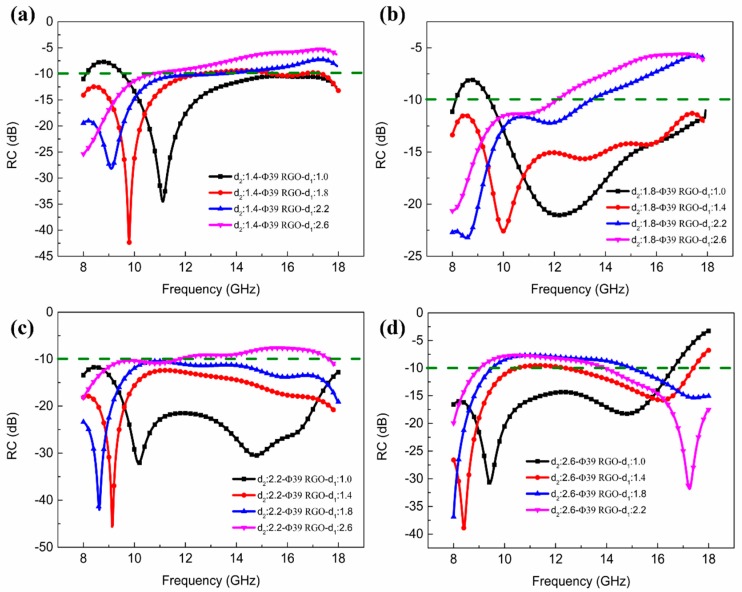
Experimental results of RC of the composites with RGO-800 films in periodic array for different thicknesses of the FR4 laminates (**a**) *d*_2_ = 1.4 mm; (**b**) *d*_2_ = 1.8 mm; (**c**) *d*_2_ = 2.2 mm; (**d**) *d*_2_ = 2.6 mm.

The RC of the composites containing RGO-800 FSS films for different FR4 laminate thicknesses, is shown in Figure 5. It is evident that RC is affected by the thickness of the upper and lower FR4 layer. RC_min_ gradually shifts towards the lower frequencies with increasing laminate thickness. For *d*_1_ = 1.0 mm and *d*_2_ = 2.2 mm, the composite exhibits excellent microwave absorption performance, with EAB spanning the entire band from 8 to 18 GHz with an average RC of −22.8 dB, as shown in Figure 5c. When *d*_1_ = 1.4 mm and *d*_2_ = 2.2 mm, the composite achieves RC_min_ of −46 dB with EAB of 10 GHz. Additionally, the composite with *d*_1_ = 1.4 mm and *d*_2_ = 1.8 mm in Figure 5c also has a wide EAB with 10 GHz.

**Figure 6:**
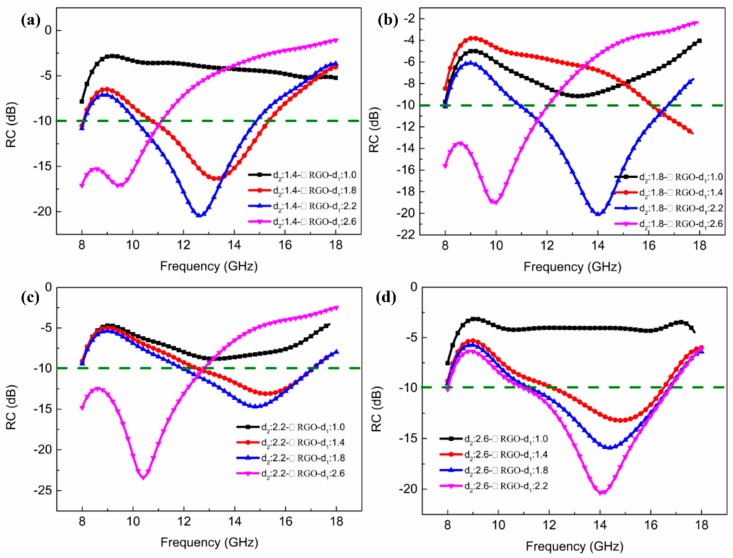
Experimental results of RC of the composites with RGO-800 films in full coverage for different thicknesses of the FR4 laminates (**a**) *d*_2_ = 1.4 mm; (**b**) *d*_2_ = 1.8 mm; (**c**) *d*_2_ = 2.2 mm; (**d**) *d*_2_ = 2.6 mm.

To verify that RGO-800 FSS films play a very important role in absorbing microwave energy, composites with full coverage between the FR4 laminates were analyzed, as shown in Figure 6. For *d*_1_ = 2.2 mm, *d*_2_ = 1.8 mm, the composite has the widest EAB of 5.9 GHz ranging from 10.9 GHz to 16.8 GHz and RC_min_ = −20.1 dB, as shown in Figure 6b.

**Figure 7:**
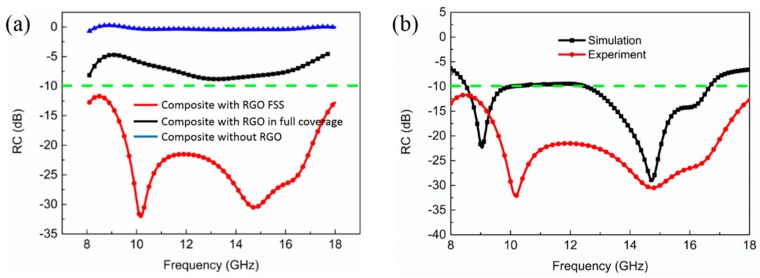
(**a**) RC of the composite with RGO-800 FSS films, RGO-800 in full coverage and without RGO-800, respectively; (**b**) Comparison of RC between numerical simulation and experimental results for the composite with RGO-800 FSS (*d*_1_ = 1.0 mm, *d*_2_ = 2.2 mm).

The composites with *d*_1_ = 1.0 mm and *d*_2_ = 2.2 mm in Figure 5c and Figure 6c along with the composite without RGO-800 are chosen for further analysis, as shown in Figure 7a. It can be observed that the composite with RGO-800 FSS films covers the entire X and Ku band, whereas the composite with RGO-800 film in full coverage has a much narrower EAB. In contrast, the composite without RGO-800 film has almost no microwave absorption ability establishing that the introduction of periodic RGO films into a sandwich structure could effectively enhance their microwave absorption properties. Figure 7b shows the simulation result of the composite with RGO-800 FSS films comparing with the optimal experimental result (*d*_1_ = 1.0 mm, *d*_2_ = 2.2 mm). There is a similar tendency between the simulated and experimental results. Although it is difficult to eliminate the difference between the two results, it can be reduced by optimizing algorithm and refining database as the cases in other studies [21,22,23].

### 3.3. Absorption Mechanism of the Composites

**Figure 8:**
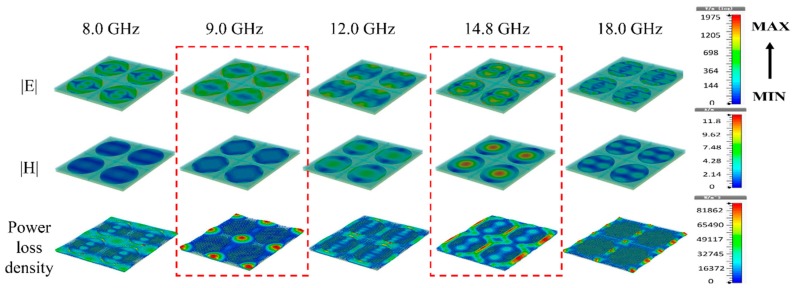
The electric, magnetic field and power loss density distributions on the composite at different frequencies.

On a macroscopic scale, the upper FR4 layer of the composite has a relatively smaller permittivity, which improves the impedance matching and reduces the microwave reflection. Combining RGO FSS with the sandwich structure can bring about impedance matching and lead to enhanced microwave absorbance and bandwidth. Based on equivalent circuit model and transmission line theory, when the thickness *d* of the structure is set, the ideal *Rs* of the RGO FSS for perfect absorption is frequency dependent. For the composite (*d*_1_ = 1.0 mm, *d*_2_ = 2.2 mm) with RGO-800 FSS films, two resonant peaks at 10.0 GHz and 14.7 GHz are excited. The electric field, magnetic field and power loss density distributions for the composite at different frequencies have been performed by commercial CST, as shown in Figure 8. As an electrically lossy material, the power loss distribution is similar to that of the electric field at the two resonant peaks of 9.0 GHz and 14.8 GHz. Due to electromagnetic coupling effects, there is strong microwave absorption between the adjacent unit cells at two resonant peaks as proved in Figure 8, which is different from the case in full coverage situation.

**Figure 9:**
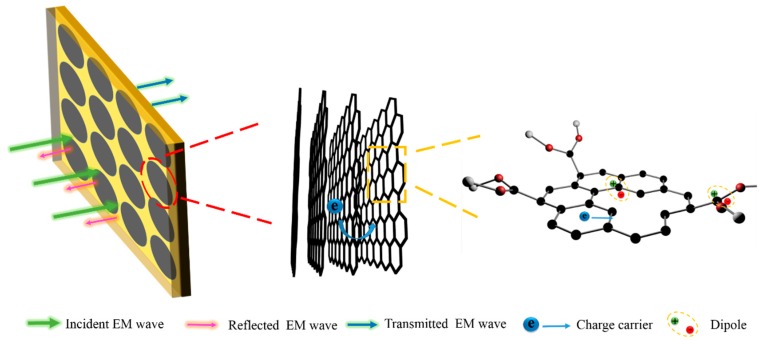
Schematic for absorption mechanism of the sandwich-structured composite in micro scale.

On a microscopic scale, RGO films are composed of layered graphene sheets that are parallel to the substrate, as is evident from the SEM image in Figure 3a. When the electromagnetic waves are incident in the vertical direction, the layered structure creates more barriers leading to a higher absorption of the microwave energy [24]. Unlike traditional FSS materials such as carbon black and metal, RGO inherently has superior properties. One the one hand (Figure 9), there are abundant micro-defects in RGO (higher D band in Raman spectrum) as compared to traditional carbon materials, which act as polarized centers by accumulating dipoles in the alternating EM field and inducing polarization loss [33,34,35]. On the other hand, due to the multilayer structure and the increase of conductivity after annealing, RGO can facilitate the hopping of charge carriers through conductive paths, which leads to considerable improvement of conduction loss [36,37].

**Figure 10:**
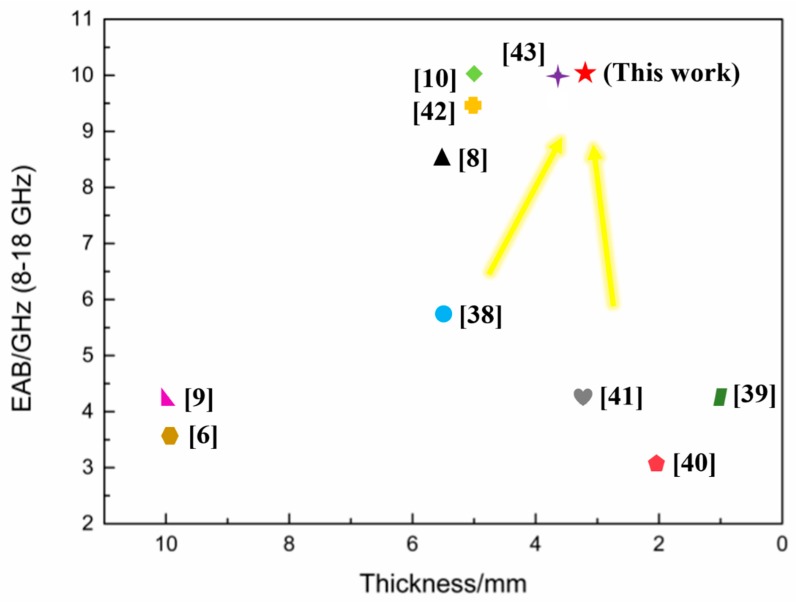
Effective absorption bandwidth (EAB) from 8–18 GHz versus thickness of typical sandwich-structured composites published in open literature.

Figure 10 summarizes the microwave absorption performance of the composites with multilayer structure in recent years. The sandwich structure combined with the FSS makes the proposed composite highly competitive with respect to other composites with pure multilayer structure, which cannot satisfy low thickness and wide bandwidth conditions simultaneously [6,9,38,39,40,41]. Introducing FSS into the sandwich structure can be an effective way to solve the above issue [10,42,43]. Li’s approach involved stencil printing of carbon black nanoparticle ink mixed with multiwalled carbon nanotubes, which is complex and suffers from the problem involving the dispersion of carbon nanotubes [10]. A similar drawback has been observed with Lee’s approach where composites with RC_min_ of −17 dB were demonstrated [43]. Overall, the procedure proposed here is simple to implement and achieves a wide absorption band of 10 GHz using a lower composite thickness of 3.2 mm and a high absorption with RC_min_ of −32 dB.

## 4. Conclusions

In summary, the composite with RGO FSS films sandwiched between two dielectric FR4 layers was fabricated using a facile approach and the effect of RGO FSS films on the microwave absorption properties of the composite was investigated. Through finite integration simulations, the composite with RGO-800 FSS films was found to exhibit excellent EM wave absorption performance with the thickness of 3.2 mm, a strong absorption with RC_min_ of −32 dB and wide EAB from 8 to 18 GHz, spanning the entire X and Ku band. Highest absorption was observed with RC_min_ of −46 dB for a total dielectric layer thickness of 3.6 mm. Thus, it was established that the composite with the multi-scale layered structure based on RGO films as FSS, possessed strong absorption and broad bandwidth while maintain a low thickness. This work provides guidelines for the fabrication of ceramic matrix composites with microwave absorbing function.

## Figures and Tables

**Table 1:** Sheet Resistance of RGO Film at Different Temperature.

Temperature/°C	Room Temperature	600	800	1000
Sheet resistance/(Ώ/sq)	3100 ± 10	70 ± 3	40 ± 3	27 ± 3
